# Risk factors of total blood loss in the posterior surgery for patients with thoracolumbar metastasis

**DOI:** 10.1186/s12891-021-04789-2

**Published:** 2021-10-22

**Authors:** Yunpeng Cui, Xuedong Shi, Chuan Mi, Bing Wang, Huaijin Li, Yuanxing Pan, Yunfei Lin

**Affiliations:** 1 Department of Orthopaedics, Peking University First Hospital, No.7 Xishiku Street, Xicheng District, Beijing, 100032 China; 2 Department of Anesthesia, Peking University First Hospital, Beijing, China

**Keywords:** Thoracolumbar, Spinal metastasis, Posterior approach, Conventional surgery, Blood loss, Gross equation

## Abstract

**Background:**

Blood loss in posterior surgery patients with thoracolumbar metastasis posed a significant challenge to surgeons. This study aimed to explore the risk factors of blood loss in posterior surgery for patients with thoracolumbar metastasis.

**Methods:**

One hundred forty-two patients were retrospectively reviewed. Their baseline characteristics were recorded. The Gross equation was used to calculate blood loss on a surgical day. Multivariate linear regression was used to analyze the risk factors.

**Results:**

Mean blood loss of 142 patients were 2055 ± 94 ml. Hypervascular primary tumor (kidney, thyroid and liver) (*P* = 0.017), wide or marginal excision (en-bloc: *P* = 0.001), metastasis at the lumbar spine (*P* = 0.033), and the presence of extraosseous tumor mass (*P* = 0.012) were independent risk factors of blood loss in the posterior surgery. Sub-analysis showed that wide or marginal excision (en-bloc: *P* **<** 0.001) and metastasis at lumbar spine (*P* = 0.007) were associated with blood loss for patients with non-hyper vascular primary tumors. Wide or marginal excision (piece-meal: *P* = 0.014) and the presence of an extraosseous tumor mass (*P* = 0.034) were associated with blood loss for patients with hypervascular primary tumors.

**Conclusion:**

Hypervascular primary tumor (kidney, thyroid, and liver) was an independent risk factor of blood loss in the posterior surgery. The presence of extraosseous tumor mass and wide or marginal excision (piece-meal) were independent risk factors for patients with hypervascular primary tumors. Metastasis at the lumbar spine and wide or marginal excision (en-bloc) were independent risk factors for patients with non-hyper vascular primary tumors.

## Background

The spine is the most common site of bone metastasis. The spine is the most common site of bone metastasis. The number of patients with spinal metastasis has increased significantly due to a growing population and prolonged survival of cancer patients [1]. Intractable pain and neurological dysfunction caused by spinal metastasis seriously affect patients’ quality of life. Some studies apply a new technique to treat these patients, such as minimally invasive surgery [2]. However, posterior surgery is still the first choice for rapidly progressing spinal cord and nerve root compression [3, 4].

Blood loss is a significant problem for patients with spinal metastasis receiving posterior surgery. A meta-analysis showed that the mean intraoperative blood loss of patients with spinal metastasis was 2180 ml [5], and the amount of blood loss had been proven to be closely related to the occurrence of perioperative complications [6]. Blood loss during surgery could be divided into dominant and recessive. The dominant blood loss mainly comes from tumor wounds, expanded epidural venous plexus, and cancellous bone surface, while recessive blood loss is mainly related to interstitial oozing and hemolysis. Some previous studies had explored the risk factors of blood loss of posterior surgery for patients with spinal metastasis but only based on the amount of blood loss recorded during the surgery, ignoring the amount of recessive blood loss [7–9]. The concept of recessive blood loss was popular among spinal and joint degeneration diseases. The mean recessive blood loss for patients with spinal degeneration was accounted for about 50% of the total blood loss and seriously affects patients’ postoperative rapid recovery [10]. There was still a lack of study about the total blood loss (dominant and recessive) posterior surgery for patients with spinal metastasis.

This study conducted a retrospective analysis of patients with thoracolumbar metastasis treated in our department. The Gross equation [11] was used to calculate the total blood loss on a surgical day. This study aimed to help the surgeon identify total blood loss risk factors on a surgical day. It is of great significance to identify patients at high risk of more blood loss and take necessary countermeasures to ensure patients’ safety and rapid recovery and reduce perioperative complications.

## Methods

### Study design and selection criteria

Inclusion: This was a single-centered retrospective study of patients with thoracolumbar metastasis who underwent posterior surgery in our department from January 2011 to December 2017. A total of 170 patients were included as our initial cases.

Exclusion: We excluded those patients with hematological tumors (19 cases), missing imaging data (5 cases) or laboratory tests (1 case), and primary tumors of unknown sources (3 cases). At last, 142 patients were included in this study.

**Fig. 1:**
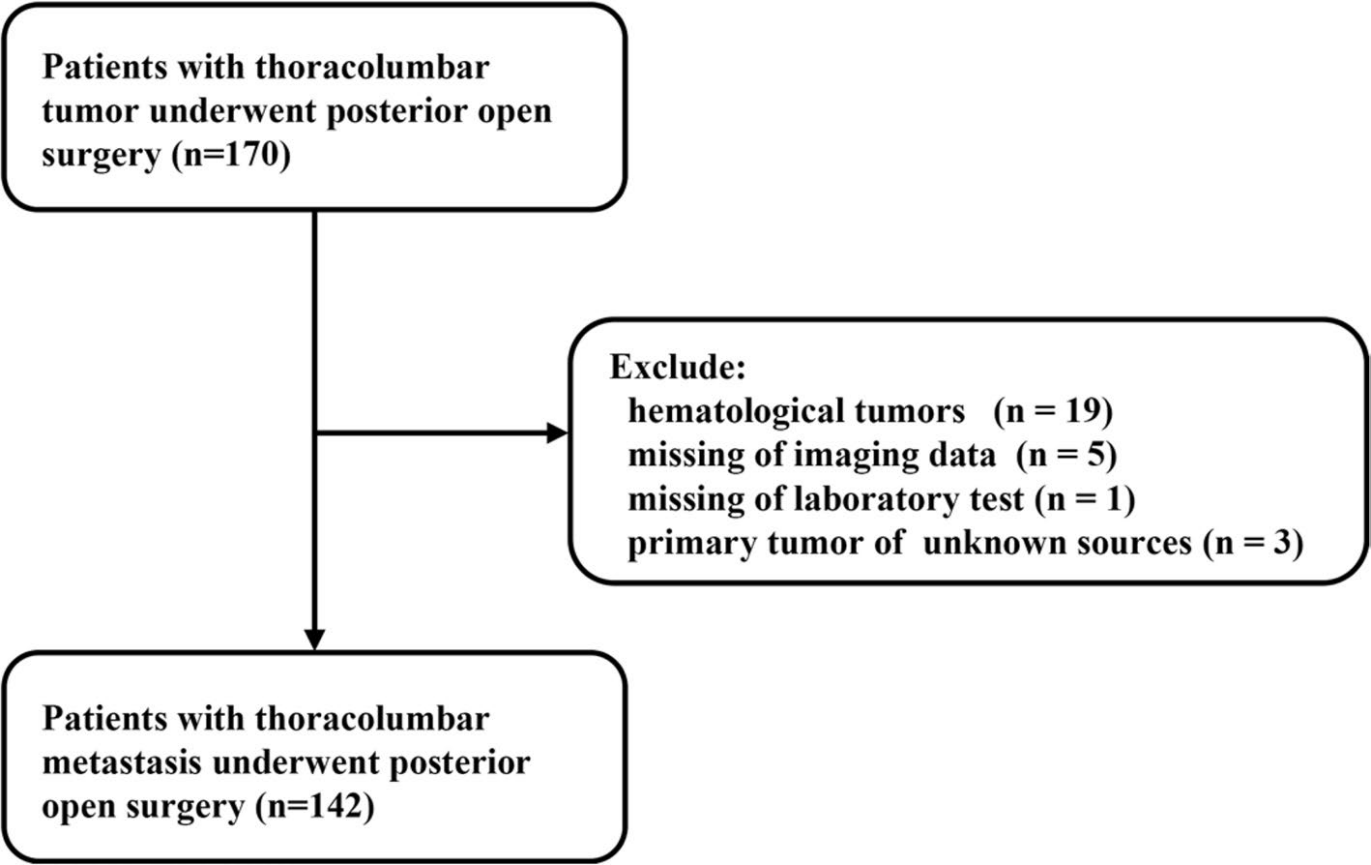
The flowchart of patient inclusion

All patients underwent a physical examination. X-ray and Magnetic Resonance Imaging (MRI) were performed to confirm the lesion’s location. Their blood samples were taken before surgery for a routine test. Surgical indications were intractable pain due to spinal instability and myelopathy caused by spinal cord compression. The surgery option was determined by multidisciplinary cooperation, an experienced neuro-radiologist, a spinal tumor surgeon, and an oncologist. Our institute’s research ethics boards approved the study protocol and required neither patient approval nor informed consent to review patients’ images and medical records. Moreover, this study was conducted following the declaration of Helsinki. The flow of patient enrolment is shown in Fig. 1.

The Gross equation calculated the total blood loss on a surgical day.

Gross equation: total perioperative blood loss = theoretical total blood loss + allogeneic blood transfusion (the patients in this study did not use autologous blood transfusion during and after surgery)

$$\mathrm{Theoretical}\ \mathrm{total}\ \mathrm{blood}\ \mathrm{loss}=\mathrm{estimated}\ \mathrm{blood}\ \mathrm{volume}\times 2\times \left(\mathrm{preoperative}\ \mathrm{Hct}-\mathrm{postoperative}\ \mathrm{Hct}\right)/\left(\mathrm{preoperative}\ \mathrm{Hct}+\mathrm{postoperative}\ \mathrm{Hct}\right)$$

[12]

$$\mathrm{Patient}'\mathrm{s}\ \mathrm{estimated}\ \mathrm{blood}\ \mathrm{volume}=\mathrm{k}1\times \mathrm{height}\ {\left(\mathrm{m}\right)}^3+\mathrm{k}2\times \mathrm{weight}\ \left(\mathrm{kg}\right)+\mathrm{k}3$$

$$\mathrm{Male}\ \mathrm{patients}\ \mathrm{k}1=0.3669,\mathrm{k}2=0.03219,\mathrm{k}3=0.6041$$

$$\mathrm{Female}\ \mathrm{patients}\ \mathrm{k}1=0.3561,\mathrm{k}2=0.03308,\mathrm{k}3=0.1833$$

### Data collection

Among the clinical variables, we reviewed the medical record to assess demographic information, BMI (non-overweight < 25; overweight: ≥25), tumor-related information, surgery-related information, and HGB before the operation. Tumor-related information included primary type. The Blood supply of primary cancer was divided into hypervascular (liver cancer, kidney cancer, thyroid cancer) or non-hyper vascular [13]. Surgery-related information included surgical methods (palliative surgery: piece-meal resection, wide or marginal excision: piece-meal or en-bloc resection), surgical site (upper thoracic spine: T1-T6, lower thoracic spine: T7-T12, lumbar spine: L1-L5), the number of surgery segments (single, multiple), the presence of extraosseous tumor mass, and preoperative embolism.

Surgical techniques and typical cases.

**Fig. 2:**
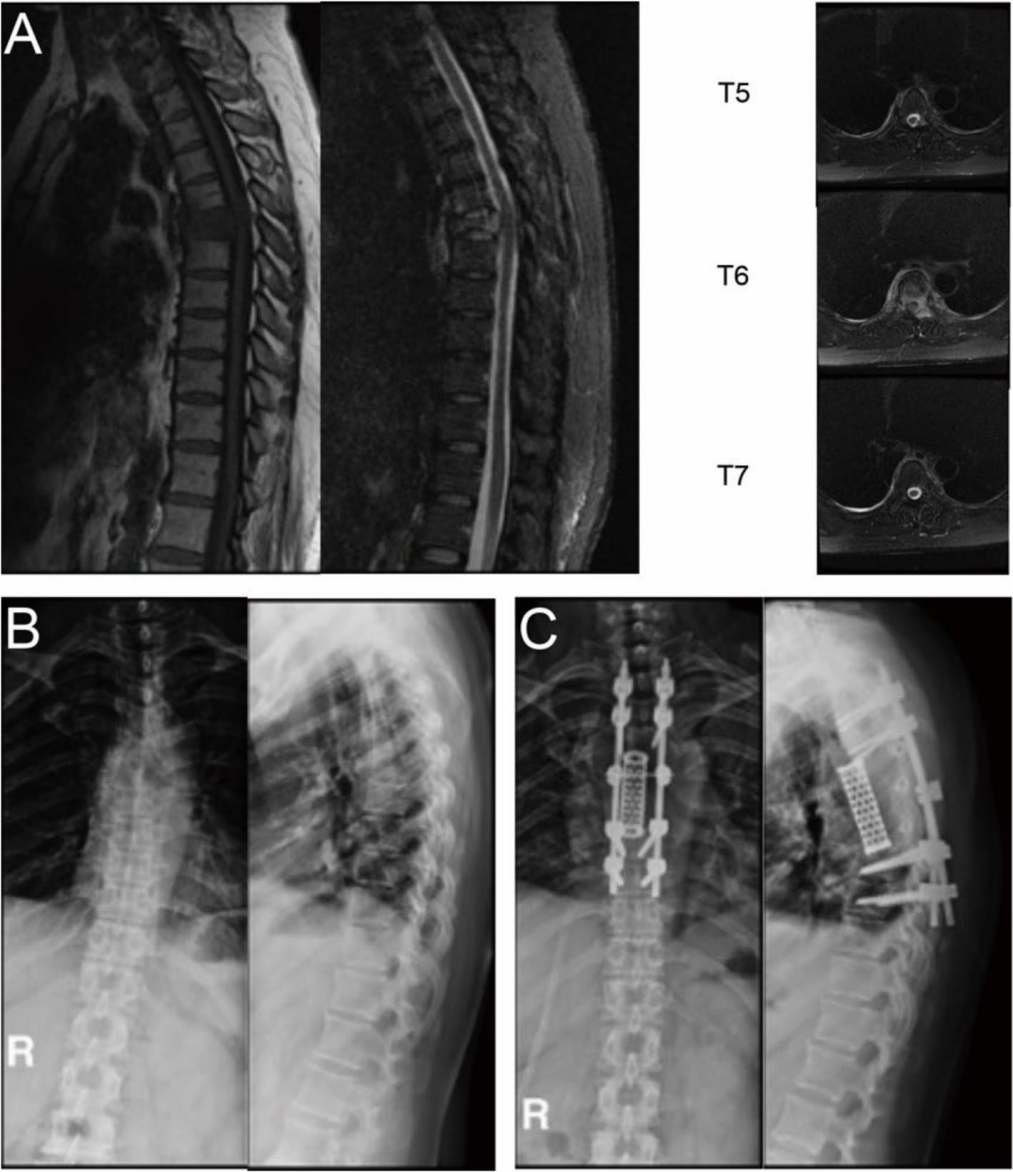
A 49-year-old woman with spine metastases of breast cancer. The patient underwent posterior marginal excision (en-bloc). The total blood loss was 4121 ml. **A** Preoperative sagittal thoracic vertebral MRI showed vertebral collapse at T6. Preoperative transversal MRI showed that the tumor involved the upper and lower adjacent vertebral bodies (T5 and T7); **B** Preoperative anteroposterior thoracic vertebral radiography; **C** Postoperative lateral anteroposterior thoracic vertebral radiography

**Fig. 3:**
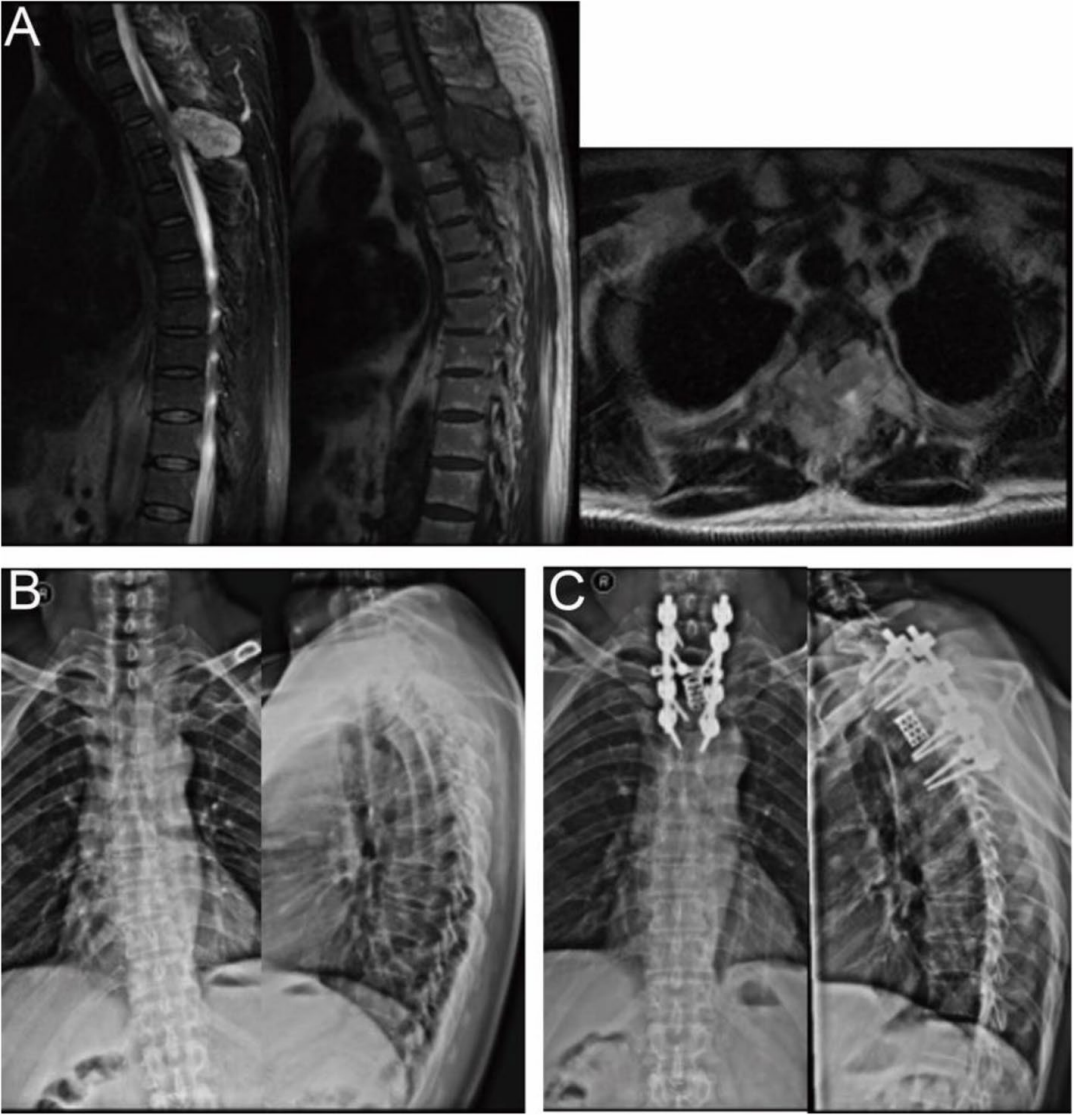
A 57-year-old man with spine metastases of renal cell carcinoma. The patient underwent preoperative arterial embolism and posterior marginal excision (piece-meal). The total blood loss was 7429 ml. **A** Preoperative sagittal thoracic vertebral MRI showed vertebral collapse at T3. Preoperative transversal MRI showed an extraosseous tumor mass at T3; **B** Preoperative anteroposterior thoracic vertebral radiography; **C** Postoperative lateral anteroposterior thoracic vertebral radiography

Experienced spinal surgeons under general anesthetic performed all operations. Surgery was performed via the median posterior approach, and paravertebral muscles were stripped to expose the lamina and facet joints. Pedicle screws were usually inserted into two levels of the upper and lower vertebra. Palliative surgery, wide or marginal excision, was performed. The intraoperative blood transfusion was considered when HGB dropped lower than 80.0 g/L. Typical cases are showed in Figs. 2 and 3.

### Statistical analysis

Data results were analyzed with SPSS 25.0 statistical software (IBM Corporation, Armonk, NY.). Continuous variables were expressed as mean (standard deviation), and categorical variables were expressed as numbers. Differences were tested using one-way ANOVA between different groups. Multivariate analysis was performed by linear regression using a stepwise approach to identify independent predictors of blood loss in the posterior surgery. Partial correlation was used to analyze the related factors of blood transfusion. *P* <  0.05 was considered as statistically significant (two-sided test).

## Results

### Patient ‘s baseline characteristics and blood loss

The study included 142 patients with a mean age of 60.9 ± 0.9 years. There were 99 males and 43 females. The Mean blood loss of 142 patients was 2055 ± 94 ml. The mean blood loss of patients with primary tumors of different blood supply was divided as shown in Table 1. The patient’s baseline characteristics and feature-related mean blood loss are shown in Table 2. One-way ANOVA was used to detect the difference in mean blood loss between characteristics. Patients with hypervascular primary tumors (kidney, thyroid, and liver) had significantly more mean blood loss than other types of tumor (*P* = 0.004). Patients with extraosseous tumor mass (*P* = 0.035) and who underwent wide or marginal excision (*P* <  0.001) had significantly more mean blood loss than other patients.

**Table 1:** Mean blood loss of different tumor

Item	N	Blood loss (ml)
Hypervascular		
Kidney	29	2599 ± 269
Liver	11	2269 ± 329
Thyroid	3	2827 ± 692
**Subtotal**	43	2531 ± 203
Non-hyper vascular		
Lung	43	1755 ± 135
Urothelial	4	2877 ± 525
Prostate	23	1730 ± 157
Breast	15	2085 ± 260
colorectal	9	2045 ± 493
Cervical	3	945 ± 85
Myxoliposarcoma	2	1833 ± 8
**Subtotal**	99	1848 ± 96
**Total**	142	2055 ± 94

**Table 2:** Comparison of blood loss between different baseline characteristics

	N	Mean Blood loss(ml)	*P*
BMI(kg/m2)			0.706
Overweight	53	2101 ± 139	
Non-Overweight	89	2027 ± 126	
Primary tumor			**0.004**
Non-hyper vascular	99	1848 ± 96	
Hypervascular	43	2531 ± 203	
Surgical method			**< 0.001**
Palliative surgery (piece-meal)	126	1936 ± 83	
Wide or marginal excision (piece-meal)	11	2670 ± 623	
Wide or marginal excision (en-bloc)	5	3710 ± 598	
Surgical site			0.525
Upper thoracic	56	2022 ± 164	
Llower thoracic	44	1945 ± 157	
Lumbar	42	2213 ± 163	
Surgery segment			0.783
Single	59	2086 ± 175	
Multiple	83	2033 ± 104	
Extraosseous tumor mass			**0.035**
No	104	1936 ± 97	
Yes	38	2382 ± 226	
Preoperative embolism			0.051
No	130	1999 ± 90	
Yes	12	2658 ± 526	
PLT (10^9^/L)			0.251
< 100	6	2570 ± 552	
≥ 100	136	2032 ± 95	

### Factors affecting blood loss on a surgical day

Univariate linear analysis showed that hypervascular primary tumor (*P* = 0.001, β = 683), wide or marginal excision (piece-meal: *P* = 0.001, β = 735; en-bloc: *P* <  0.001, β = 1775) and the presence of extraosseous tumor mass (*P* = 0.035, β = 446) were significantly associated with average blood loss. Further multivariate linear analysis showed that four factors were independent risk factors for blood loss in the posterior surgery. These factors included hypervascularprimary tumor (*P* = 0.017, β = 529), wide or marginal excision (en-bloc: *P* <  0.001, β = 2053), metastasis at lumbar spine (*P* = 0.033, β = 470), and the presence of extraosseous tumor mass (*P* = 0.012, β = 513) Table 3.

**Table 3:** Linear regression analysis of risk factors for blood loss

	Univariate analysis		Multivariate analysis	
	*Regression Coefficient*	*P*	*Regression Coefficient*	*P*
BMI (kg/m2)				
Overweight	74	0.706	172	0.340
Blood supply				
Hypervascular	683	**0.001**	529	**0.017**
Surgical method				
Wide or marginal excision (piece-meal)	735	**0.030**	641	0.059
Wide or marginal excision (en-bloc)	1775	**< 0.001**	2053	**< 0.001**
Surgical site				
Lower thoracic	−77	0.734	106	0.623
Lumbar	191	0.408	470	**0.033**
Surgery segment				
Multiple	−53	0.783	21	0.907
Extraosseous tumor mass				
Yes	446	**0.035**	513	**0.012**
Preoperative embolism				
No	− 660	0.051	− 159	0.666
PLT (10^9^/L)				
< 100	539	0.251	614	0.166

The sub-analysis showed that wide or marginal excision (en-bloc: *P* < 0.001, β = 2400) and metastasis at lumbar spine (*P* = 0.007, β = 619) were significantly associated with blood loss for patients with non-hypervascular primary tumor. For patients with hypervascular primary tumor, wide or marginal excision (piece-meal: *P* = 0.014, β = 1926) and the presence of extraosseous tumor mass (*P* = 0.034, β = 913) were significantly associated with blood loss in the posterior surgery Table 4.

**Table 4:** Multivariate linear regression analysis of risk factors for tumors with different blood supply

Primary tumor type	Non-hyper vascular		Hypervascular	
	*Regression Coefficient*	*P*	*Regression Coefficient*	*P*
BMI (kg/m2)				
Overweight	190	0.307	54	0.905
Surgical method				
Wide or marginal excision (piece-meal)	123	0.727	1926	**0.014**
Wide or marginal excision (en-bloc)	2400	**< 0.001**	1596	0.106
Surgical site				
Lower thoracic	−3	0.990	553	0.288
Lumbar	619	**0.007**	38	0.943
Surgery segment				
Multiple	134	0.478	−175	0.683
Extraosseous tumor mass				
Yes	282	0.205	913	**0.034**
Preoperative embolism				
No	NA	NA	207	0.701
PLT (10^9^/L)				
< 100	553	0.386	515	0.468

## Discussion

Patients with spinal metastasis have significant differences in individual blood loss during posterior surgery. The study of Kumar N et al. showed that the mean blood loss in posterior surgery for patients with metastasis was only 870 ± 720 ml [9], while most studies found that patients would suffer more blood loss during surgery. The Meta-analysis of Chen Y et al. showed that the mean intraoperative blood loss in posterior surgery for patients with metastasis was 2180 ml, and 12% of patients exceeded 5000 ml [5]. Gao X et al.’s study is roughly the same as Chen Y et al.’ study, with the mean blood loss of 1756 ± 1218 ml [8]. Although the amount of recessive blood loss during posterior surgery for patients with spinal metastasis was still uncertain, the amount of blood loss in the previous studies was based on the operation record, ignoring the amount of recessive blood loss and might underestimate the patient’s total blood loss.

We carried out this retrospective study, and the Gross equation was used to calculate the total blood loss on a surgical day. The results showed that the mean blood loss was 2055 ± 94 ml on the surgical day, similar to Chen Y et al. and Gao X et al.’s study. Hypervascular primary tumor (kidney, thyroid, and liver), wide or marginal excision, metastasis at the lumbar spine, and the presence of extraosseous tumor mass were significantly associated with blood loss. The results were partially consistent with previous studies except for the surgical method. Gao X et al.’s study showed that the risk factors for intraoperative blood loss in patients with spinal metastasis, including the primary tumor type, tumor location, exposed segment, decompression segment, and surgical method, in which En bloc resection had less intraoperative blood loss compared with piece-meal resection [8]. En bloc resection was one kind of wide or marginal excision, requiring extensive tissue separation. En bloc excision would reduce the intraoperative blood loss from tumor wounds independent of the primary tumor type. However, the extensive separation of normal tissue and longer surgery time would significantly increase the interstitial oozing (recessive blood loss). Patients who underwent palliative surgery were not the same as patients who underwent wide or marginal excision. Recessive blood loss consisted of a small part of total blood loss due to the more considerable amount of intraoperative blood loss. Based on our and previous study results, recessive blood loss had different effects in different surgical procedures. The surgeon should pay special attention to patients who underwent wide or marginal excision for massive recessive blood loss.

Patients with hypervascular primary tumors had significantly more blood loss than patients with other types in this study. Many studies confirmed preoperative embolization for patients with hypervascular primary tumors that could effectively reduce intraoperative blood loss [14, 15]. The present study did not find a correlation between preoperative embolization and blood loss. First, this might be related to the selection bias. Only 10 patients with renal cancer and 2 patients with liver cancer received preoperative embolization in our study. Second, the inclusion of recessive blood loss might weaken the effect of embolization on the control of bleeding. Preoperative embolization for spinal tumors had a risk of spinal ischemia [16]. Selective computed tomography angiography for detecting radiculomedullary arteries was an effective method to find radiculomedullary arteries and improve security [17].

Intraoperative blood loss for patients with spinal metastasis mainly comes from tumor wounds, especially those with hypervascular primary tumors. For these patients, the presence of extraosseous tumor mass significantly increased the intraoperative bleeding. It was necessary to remove all tumor tissue to control the bleeding from tumor wounds when patients underwent piece-meal resection. For patients with hypervascular primary tumors and who underwent piece-meal resection, proper stripping in normal tissues to minimize the blood supply of tumor masses could effectively reduce the intraoperative from tumor wounds. Treatment with bipolar electrocoagulation before piece-meal resection of tumor mass could also effectively reduce the intraoperative wound bleeding.

Tang X et al. reviewed 173 patients with sacral tumors. The results showed that tumor volume greater than 200 cm3 was a risk factor for more blood loss [18]. Tumor volume greater than 5 cm was considered a risk factor for excessive blood loss for bone tumors [19]. The volume of the lumbar spine is more significant than that of the upper thoracic spine [20], and the presence of extraosseous tumor mass also increases the tumor volume. Otherwise, the lumbar spine’s height is higher than that of the thoracic spine, so the surgical incision is longer than that of the thoracic spine, which increases the dissection range and interstitial space, and a result of more recessive blood loss in the interstitial space.

Recently, some surgeons have tried to apply minimally invasive spine surgery for patients with spinal metastasis. Minimally invasive spine surgery is performed through physiological tissue gaps. Less tissue stripping can effectively reduce intraoperative bleeding and postoperative interstitial oozing. Chou D et al. and Zhu X et al. showed that minimally invasive spine surgery could reduce intraoperative blood loss and postoperative drainage compared with conventional surgery [21, 22]. Even for patients who underwent en-bloc, minimally invasive spine surgery also had an advantage compared with conventional surgery [23]. Minimally invasive spine surgery might be a good choice characterized by less physiological insult and fewer postoperative complications, allowing early mobilization and rapid recovery.

There are limitations to the present study. First, it was limited by its retrospective nature, and there would be a particular bias in patient selection. Second, the period of this study was relatively long, and the learning curve of surgical skills was not included in the factors analyzed in this study. Last, for the relatively long-time span of the present study, the anesthesia protocol was not completely consistent during the study. This would have an impact on blood bleeding. However, this study provides important information regarding total blood loss risk factors in conventional surgery patients with thoracolumbar metastasis on a surgical day.

## Conclusions

Hypervascular primary tumor (kidney, thyroid, and liver) was an independent risk factor of blood loss in the posterior surgery. The presence of extraosseous tumor mass and wide or marginal excision (piece-meal) were independent risk factors for patients with hypervascular primary tumor. Metastasis at the lumbar spine and wide or marginal excision (en-bloc) were independent risk factors for patients with non-hyper vascular primary tumors.

## Data Availability

The datasets generated and/or analysed during the current study are not publicly available due privacy or ethical restrictions but are available from the corresponding author on reasonable request.
